# Supplementary material for: Epibiotic Fungal Communities of Three Tomicus spp. Infesting Pines in Southwestern China
Source: Microorganisms. 2019 Dec 20;8(1):15. doi: 10.3390/microorganisms8010015 (PMC7023379; doi:10.3390/microorganisms8010015)
Supplement: Supplementary file 1 [file microorganisms-08-00015-s001.zip › Supplementary Materials/Supplementary Figure S1.pdf]

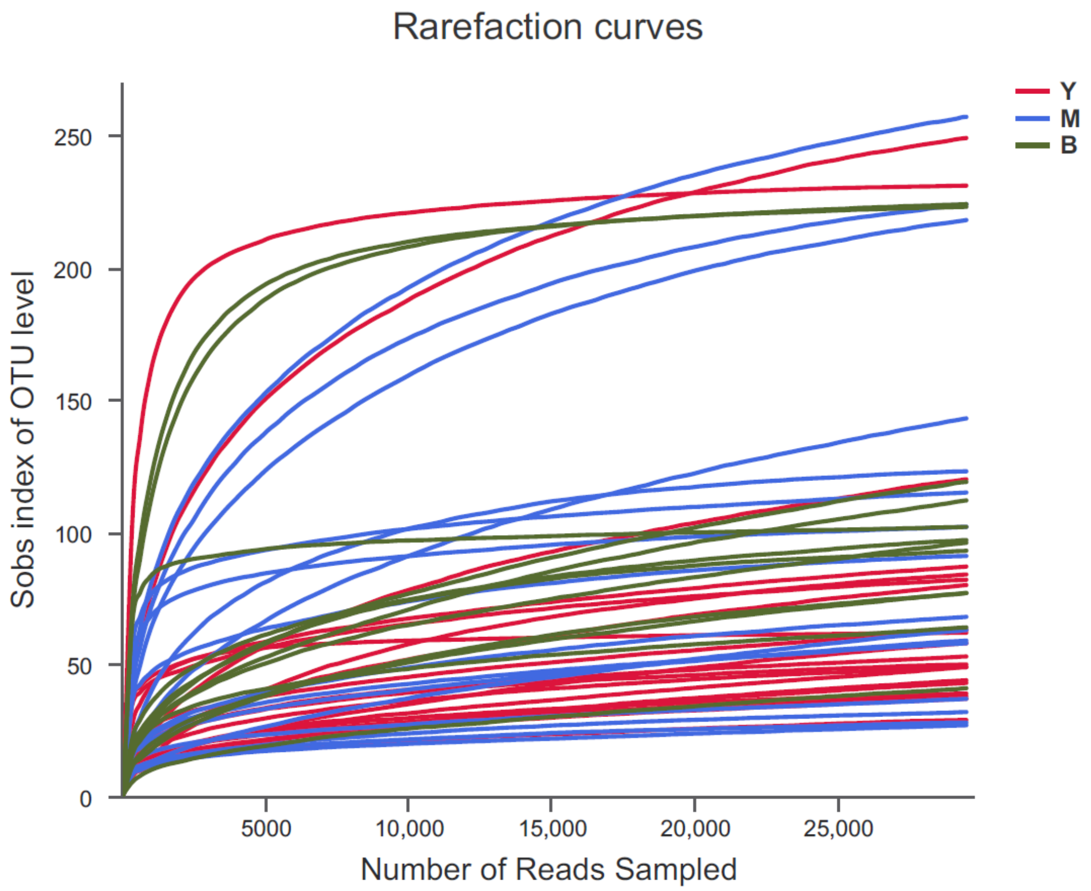

Supplementary Figure S1| Rarefaction curves of the 48 samples. Different colors represent distinct bark beetles.
Y = *T. yunnanensis*; M = *T. minor*; B = *T. brevipilosus*.
