# Supplementary material for: Epibiotic Fungal Communities of Three Tomicus spp. Infesting Pines in Southwestern China
Source: Microorganisms. 2019 Dec 20;8(1):15. doi: 10.3390/microorganisms8010015 (PMC7023379; doi:10.3390/microorganisms8010015)
Supplement: Supplementary file 1 [file microorganisms-08-00015-s001.zip › Supplementary Materials/Supplementary-Table. 2.docx]

**Supplementary-Table 2|** Samples information used for this study.

| No. | Sample | Host | Location | Beetle | Branch\Trunk | Number of beetles |
| --- | --- | --- | --- | --- | --- | --- |
| 1 | BPEB-1 | *Pinus* *kesiya* | Puer | *Tomicus* *brevipilosus* | branch | 28 |
| 2 | BPEB-2 | *P*. *kesiya* | Puer | *T*. *brevipilosus* | branch | 29 |
| 3 | BPEB-3 | *P*. *kesiya* | Puer | *T*. *brevipilosus* | branch | 29 |
| 4 | BPEB-4 | *P*. *kesiya* | Puer | *T*. *brevipilosus* | branch | 29 |
| 5 | BPEB-5 | *P*. *kesiya* | Puer | *T*. *brevipilosus* | branch | 30 |
| 6 | TPEB-1 | *P*. *kesiya* | Puer | *T*. *brevipilosus* | trunk | 30 |
| 7 | TPEB-2 | *P*. *kesiya* | Puer | *T*. *brevipilosus* | trunk | 30 |
| 8 | TPEB-3 | *P*. *kesiya* | Puer | *T*. *brevipilosus* | trunk | 25 |
| 9 | TPEB-4 | *P*. *kesiya* | Puer | *T*. *brevipilosus* | trunk | 29 |
| 10 | BQJB-1 | *P. yunnanensis* | Qujing | *T*. *brevipilosus* | branch | 29 |
| 11 | BQJB-2 | *P. yunnanensis* | Qujing | *T*. *brevipilosus* | branch | 30 |
| 12 | TQJB-1 | *P. yunnanensis* | Qujing | *T*. *brevipilosus* | trunk | 29 |
| 13 | BQJM-1 | *P. yunnanensis* | Qujing | *T*. *minor* | branch | 27 |
| 14 | BQJM-2 | *P. yunnanensis* | Qujing | *T*. *minor* | branch | 27 |
| 15 | BQJM-3 | *P. yunnanensis* | Qujing | *T*. *minor* | branch | 27 |
| 16 | TQJM-1 | *P. yunnanensis* | Qujing | *T*. *minor* | trunk | 27 |
| 17 | TQJM-2 | *P. yunnanensis* | Qujing | *T*. *minor* | trunk | 27 |
| 18 | TQJM-3 | *P. yunnanensis* | Qujing | *T*. *minor* | trunk | 27 |
| 19 | BXYM-1 | *P. yunnanensis* | Xiangyun | *T*. *minor* | branch | 28 |
| 20 | BXYM-2 | *P. yunnanensis* | Xiangyun | *T*. *minor* | branch | 28 |
| 21 | BXYM-3 | *P. yunnanensis* | Xiangyun | *T*. *minor* | branch | 28 |
| 22 | TXYM-1 | *P. yunnanensis* | Xiangyun | *T*. *minor* | trunk | 25 |
| 23 | TXYM-2 | *P. yunnanensis* | Xiangyun | *T*. *minor* | trunk | 30 |
| 24 | BYXM-2 | *P. yunnanensis* | Yuxi | *T*. *minor* | branch | 25 |
| 25 | BYXM-3 | *P. yunnanensis* | Yuxi | *T*. *minor* | branch | 26 |
| 26 | BYXM-1 | *P. yunnanensis* | Yuxi | *T*. *minor* | branch | 26 |
| 27 | TANM-1 | *P. yunnanensis* | Anning | *T*. *minor* | trunk | 30 |
| 28 | TANM-2 | *P. yunnanensis* | Anning | *T*. *minor* | trunk | 30 |
| 29 | BQJY-1 | *P. yunnanensis* | Qujing | *T. yunnanensis* | branch | 27 |
| 30 | BQJY-2 | *P. yunnanensis* | Qujing | *T. yunnanensis* | branch | 27 |
| 31 | TQJY-1 | *P. yunnanensis* | Qujing | *T. yunnanensis* | trunk | 25 |
| 32 | BXYY-1 | *P. yunnanensis* | Xiangyun | *T. yunnanensis* | branch | 30 |
| 33 | BXYY-2 | *P. yunnanensis* | Xiangyun | *T. yunnanensis* | branch | 30 |
| 34 | BXYY-3 | *P. yunnanensis* | Xiangyun | *T. yunnanensis* | branch | 29 |
| 35 | BXYY-4 | *P. yunnanensis* | Xiangyun | *T. yunnanensis* | branch | 29 |
| 36 | TXYY-1 | *P. yunnanensis* | Xiangyun | *T. yunnanensis* | trunk | 30 |
| 37 | TXYY-2 | *P. yunnanensis* | Xiangyun | *T. yunnanensis* | trunk | 30 |
| 38 | TXYY-3 | *P. yunnanensis* | Xiangyun | *T. yunnanensis* | trunk | 26 |
| 39 | TXYY-4 | *P. yunnanensis* | Xiangyun | *T. yunnanensis* | trunk | 30 |
| 40 | BYXY-1 | *P. yunnanensis* | Yuxi | *T. yunnanensis* | branch | 26 |
| 41 | BYXY-2 | *P. yunnanensis* | Yuxi | *T. yunnanensis* | branch | 26 |
| 42 | TYXY-1 | *P. yunnanensis* | Yuxi | *T. yunnanensis* | trunk | 30 |
| 43 | TYXY-2 | *P. yunnanensis* | Yuxi | *T. yunnanensis* | trunk | 27 |
| 44 | TYXY-3 | *P. yunnanensis* | Yuxi | *T. yunnanensis* | trunk | 27 |
| 45 | TYXY-4 | *P. yunnanensis* | Yuxi | *T. yunnanensis* | trunk | 27 |
| 46 | TANY-1 | *P. yunnanensis* | Anning | *T. yunnanensis* | trunk | 29 |
| 47 | TANY-2 | *P. yunnanensis* | Anning | *T. yunnanensis* | trunk | 29 |
| 48 | TANY-3 | *P. yunnanensis* | Anning | *T. yunnanensis* | trunk | 29 |
