# Supplementary material for: Epibiotic Fungal Communities of Three Tomicus spp. Infesting Pines in Southwestern China
Source: Microorganisms. 2019 Dec 20;8(1):15. doi: 10.3390/microorganisms8010015 (PMC7023379; doi:10.3390/microorganisms8010015)
Supplement: Supplementary file 1 [file microorganisms-08-00015-s001.zip › Supplementary Materials/Supplementary-Table. 3.docx]

**Supplementary-Table 3|** Eight orders with >10,000 reads in the fungal communities associated with the 3 beetles species.

| Phylum | Class | Order | OTUs | SUM reads |
| --- | --- | --- | --- | --- |
| Ascomycota | Saccharomycetes | Saccharomycetales | 70 | 939262 |
|  | Sordariomycetes | Ophiostomatales | 105 | 102167 |
|  | Eurotiomycetes | Eurotiales | 72 | 97547 |
|  | Sordariomycetes | Hypocreales | 80 | 56444 |
|  | Dothideomycetes | Capnodiales | 138 | 49373 |
|  | Dothideomycetes | Pleosporales | 86 | 46738 |
|  | unclassified_Ascomycota | unclassified_Ascomycota | 136 | 23638 |
|  | Sordariomycetes | Sordariales | 33 | 19068 |
