# Supplementary material for: Epibiotic Fungal Communities of Three Tomicus spp. Infesting Pines in Southwestern China
Source: Microorganisms. 2019 Dec 20;8(1):15. doi: 10.3390/microorganisms8010015 (PMC7023379; doi:10.3390/microorganisms8010015)
Supplement: Supplementary file 1 [file microorganisms-08-00015-s001.zip › Supplementary Materials/Supplementary-Table. 4.docx]

**Supplementary-Table 4|** Saccharomycetales, Ophiostomatales, Eurotiales OTUs and read numbers associated with the 3 beetle species.

| Order | Genus | OTU | read SUM |
| --- | --- | --- | --- |
| Saccharomycetales | *Candida* | OTU271, OTU411, OTU417, OTU5683, OTU727, OTU892 | 487 |
|  | *Debaryomyces* | OTU723 | 4798 |
|  | *Kuraishia* | OTU258, OTU314, OTU377, OTU409 | 5232 |
|  | *Meyerozyma* | OTU874 | 28 |
|  | *Nakazawaea* | OTU265, OTU509 | 23600 |
|  | *Ogataea* | OTU474, OTU335, OTU202, OTU200, OTU214, OTU484, OTU207. OYU464, OTU293, OYU249 | 11093 |
|  | *Pichia* | OYU1371 | 155 |
|  | *Saccharomyces* | OTU603 | 3 |
|  | *Schwanniomyces* | OTU603 | 2 |
|  | *Yamadazyma* | OTU500, OTU1363, OTU476, OTU485, OTU487, OTU640, OTU461, OTU452, OTU454, OTU458, OTU459, OTU384, OTU447, OTU440, OTU443, OTU442, OTU438, OTU435, OTU436, OTU399 | 486706 |
|  | Unclassified-genus | OTU798, OTU361, OTU168, OTU239, OTU406, OTU414, OTU416, OTU468, OTU437, OTU470, OTU471, OTU501, OTU139, OTU 441, OTU444, OTU602, OTU328, OTU368, OTU486, OTU1319, OTU495, OTU305 | 407155 |
|  | *Wickerhamomyces* | OTU1015 | 3 |
| Ophiostomatales | *Ceratocystiopsis* | OTU379, OTU243 | 41 |
|  | *Graphilbum* | OTU434, OTU326 | 1286 |
|  | *Leptographium* | OTU677, OTU433 | 527 |
|  | *Ophiostoma* | OTU273,OTU272,OTU277,OTU370,OTU278,OTU375,OTU194,OTU281,OTU286,  OTU304,OTU306,OTU308,OTU353,OTU332,OTU342,OTU349,OTU348, OTU269,OTU266,OTU263,OTU294,OTU222,OTU221,OTU227,OTU224,  OTU223,OTU367,OTU255,OTU59,OTU254,OTU318,OTU317,OTU259,  OTU350,OTU354,OTU359,OTU347,OTU210,OTU217,OTU215,  OTU343,OTU327,OTU248,OTU298,OTU369,OTU363,OTU963,OTU432, OTU360,OTU338,OTU262,OTU203,OTU290,OTU296,OTU1286,OTU393, OTU391, OTU1178 | 95328 |
|  | *Sporothrix* | OTU218 | 4979 |
|  | Unclassified-genus | OTU241 | 6 |
| Eurotiales | *Aspergillus* | OTU869, OTU782, OTU918, OTU84, OTU774, OTU371, OTU789, OTU695, OTU697, OTU732, OTU763, OTU1219, OTU699, OTUT911, OTU605, OTU802 | 3427 |
|  | *Byssochlamys* | OTU656 | 8 |
|  | *Monascus* | OTU709 | 1627 |
|  | *Penicillium* | OTU170, OTU492, OTU706, OTU172, OTU174, OTU408, OTU178, OTU622, OTU187, OTU574, OTU729, OTU460, OTU613, OTU502, OTU475, OTU1338, OTU930, OTU742, OTU744, OTU941, OTU179, OTU182, OTU405, OTU1349, OTU716, OTU488, OTU483, OTU181,OTU183 | 74935 |
|  | *Sagenomella* | OTU937, OTU398 | 73 |
|  | *Talaromyces* | OTU401, OTU809, OTU173, OTU185, OTU467, OTU1007, OTU1330, OUT1014, OTU956 | 17117 |
|  | *Thermomyces* | OTU610 | 149 |
|  | *Xeromyces* | OTU701 | 74 |
|  | Unclassified-genus | OTU852, OTU1352 | 127 |
