# Supplementary figures and images for: Epibiotic Fungal Communities of Three Tomicus spp. Infesting Pines in Southwestern China
Source: Microorganisms. 2019 Dec 20;8(1):15. doi: 10.3390/microorganisms8010015 (PMC7023379; doi:10.3390/microorganisms8010015)

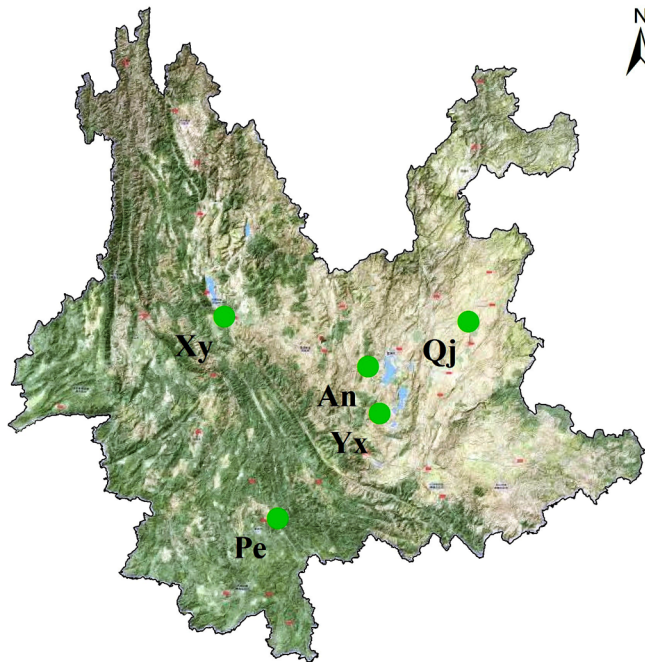

**Yunnan, China**

Supplement: Supplementary file 1 [file microorganisms-08-00015-s001.zip › Supplementary Materials/Supplementary Figure S2.pdf]
