# Supplementary material for: Epibiotic Fungal Communities of Three Tomicus spp. Infesting Pines in Southwestern China
Source: Microorganisms. 2019 Dec 20;8(1):15. doi: 10.3390/microorganisms8010015 (PMC7023379; doi:10.3390/microorganisms8010015)
Supplement: Supplementary file 1 [file microorganisms-08-00015-s001.zip › Supplementary Materials/Supplementary-Table. 1.doc]

**Supplementary-Table 1|** Base information of the sample-collection plots in Yunnan Province, China.

| Location | Host | Insect vector | longitude\latitude | altitude\m |
| --- | --- | --- | --- | --- |
| Puer,Yunnan | *P*. *kesiya* | *T*. *brevipilosus* | N22°56′36′′, E101°14′36.7′′ | 1400.7 |
| Xiangyun,Yunnan | *Pinus yunnanensis* | *Tomicus yunnanensis, T. minor* | N25°21′25′′, E100°51′49′′ | 2255.4 |
| Qujing,Yunnan | *P*. *yunnanensis* | *T*. *yunnanensis, T. minor*, *T*. *brevipilosus* | N25°28′51′′, E103°46′32′′ | 2068.2 |
| Anning,Yunnan | *P*. *yunnanensis* | *T*. *yunnanensis, T. minor* | N24°53′32′′, E102°24′23′′ | 1939.9 |
| Yuxi, Yunan | *P*. *yunnanensis* | *Tomicus yunnanensis, T. minor* | N24°18′23′′, E102°34′37′′ | 1908.1 |
